# Characterization of kallikrein-related peptidase 4 (KLK4) mRNA expression in tumor tissue of advanced high-grade serous ovarian cancer patients

**DOI:** 10.1371/journal.pone.0212968

**Published:** 2019-02-27

**Authors:** Weiwei Gong, Yueyang Liu, Christof Seidl, Tobias Dreyer, Enken Drecoll, Matthias Kotzsch, Holger Bronger, Julia Dorn, Viktor Magdolen

**Affiliations:** 1 Clinical Research Unit, Department of Obstetrics and Gynecology, Technical University of Munich, Munich, Germany; 2 Institute of Pathology, Technical University of Munich, Munich, Germany; 3 Medizinisches Labor Ostsachsen, Dresden, Germany; Universidade de Sao Paulo Instituto de Quimica, BRAZIL

## Abstract

Overexpression of several members of the kallikrein-related peptidase (KLK) family, including KLK4, has been reported in ovarian cancer tissue, consistent with the fact that elevated levels of KLK protein are often also found in serum and in effusion fluids of ovarian cancer patients. In the present study, we quantitatively analyzed KLK4 tumor tissue mRNA expression levels in a homogeneous cohort including 138 patients of advanced high-grade serous ovarian cancer (FIGO stage III/IV). Age as well as ascites fluid volume were found to be significantly associated with KLK4 mRNA expression levels. In univariate Cox regression analysis, the clinical factors residual tumor mass and ascites fluid volume represented univariate predictors for both overall survival (OS) and progression-free survival (PFS). Furthermore, elevated KLK4 mRNA expression levels were significantly linked with reduced OS (p = 0.001), but not with PFS. The results concerning the association of KLK4 mRNA expression with OS were validated in a publicly available Affymetrix-based mRNA data set from The Cancer Genome Atlas (n = 252) applying the Kaplan-Meier Plotter tool (p = 0.047). In multivariable analyses, elevated KLK4 mRNA values turned out as an additional, independent predictive marker for shortened OS (p = 0.006), whereas residual tumor mass, but not ascites fluid volume, remained an independent indicator for both OS and PFS (p < 0.001 and p = 0.002, respectively). The results of the present study, obtained in a well-defined, homogenous cohort of patients afflicted with advanced high-grade serous ovarian cancer, are in line with previous reports describing high KLK4 levels as an unfavorable marker in ovarian cancer patients.

## Introduction

Epithelial ovarian cancer still is the leading cause of death among patients with gynecological malignancies [1]. Due to the lack of obvious symptoms, approximately 75% of patients are diagnosed at advanced stages of epithelial ovarian cancer (International Federation of Gynecology and Obstetrics [FIGO] stage IIb-IV) [2], with 5-year survival rates of 17–36% [3]. Moreover, the poor prognosis of the patients afflicted with ovarian cancer originates from inefficient primary debulking surgery and rapid development of chemo-resistance [4]. Therefore, valid tumor markers for prognosis and prediction of therapy response are urgently needed.

The family of the human kallikrein-related peptidases (KLKs) comprises fifteen homologous secreted serine endopeptidases (KLK1 –KLK15), which are encoded within the largest protease-encoding gene cluster on chromosome 19q13.3–4 around the active site. All KLK genes are highly conserved in terms of exon number and exon/intro phases. On the protein level, the members of the human KLK family show a high degree of structural similarity as well [5,6]. Accumulating reports indicate that KLKs are dysregulated in many human solid tumors, especially in hormone-dependent cancers e.g. ovarian, breast and prostate cancer [7–11]. Moreover, KLKs are considered as prognostic and predictive biomarkers for various malignancies. Elevated KLK4 and KLK7 expression levels are obviously involved in paclitaxel resistance in ovarian cancer [12–14]. Overexpression of KLK10 is associated with an unfavorable prognosis and promoted tamoxifen resistance in breast cancer [15]. In ovarian cancer, expression of the majority of KLKs are not only dysregulated in cancer cells, but many of the corresponding proteins are detected in serum and in effusion fluids from patients, suggesting that KLKs are involved in the course of the disease [6,16–20]. In ovarian cancer, high levels of KLK4-7, 10 and 13 function as unfavorable indicators for prognosis, while elevated expression of KLK8, 9, 11, 14 and 15 are supposed to be favorable markers [13,21–26].

KLK4 has been shown to be expressed in ovarian cancer tissue and cell lines, but is absent in non-malignant ovarian tissues [27,28]. As reported by Obiezu and co-workers [29], a significant association was observed between elevated KLK4 mRNA levels and clinical stage as well as tumor grade. Furthermore, overexpression of KLK4 induced proliferation and increased the risk for relapse and death in ovarian carcinoma patients [13,29]. The results of these reports suggest that KLK4 may represent a valuable biomarker in diagnosis and treatment of ovarian cancer.

However, in the studies described, rather heterogeneous patient cohorts were analyzed, encompassing different histological subtypes such as low and high grade serous, mucinous as well as endometroid ovarian tumors [13,27,29]. In contrast, in the present study, a well-defined homogeneous cohort was investigated, including patients with advanced high-grade serous ovarian cancer FIGO stage III/IV only. We determined the expression levels of KLK4 mRNA by quantitative polymerase chain reaction (qPCR) and analyzed the association of KLK4 mRNA levels with representative clinical parameters as well as with survival of the patients.

## Materials and methods

### Patients and sample collection

We analyzed tumor tissue specimens of 138 advanced stage high-grade serous ovarian cancer patients which were collected from the biobank of the Department of Obstetrics and Gynecology and the Institute of Pathology (which is part of the biobank of the Klinikum rechts der Isar, TU Munich, Germany), from 1990 to 2012. The study was approved by the local Ethics Committee (Faculty of Medicine, Technical University Munich, Ismaninger Str. 22, 81675 Muenchen, Germany, ethikkommission@mri.tum.de; project 491/17 S). Written informed consent was obtained from all patients. All patients were treated with standard stage-related primary radical debulking surgery. Patients’ age at the time of surgery ranged from 33 to 88 years with a median age of 64 years. For 70 of them, all macroscopically visible tumor manifestations could be optimally removed by surgery. All patients received systemic adjuvant treatment after surgery, including platinum-based chemotherapy. In no case, neoadjuvant chemotherapy was applied. Clinical factors were archived at the time of surgery including age, ascites fluid volume and residual tumor mass. The follow-up time of patients after primary tumor resection was between 2 to 279 months for overall survival (OS; median: 29 months) and between 3 to 279 months for progression-free survival (PFS; median: 20 months).

### RNA extraction and first-strand cDNA preparation

The qPCR assay for KLK4 was established using ovarian cancer OV-MZ-6 cells overexpressing KLK4. Total RNA was isolated from this cell line using the RNeasy Mini Kit (Qiagen, Hilden, Germany), following the manufacturer’s instructions [25].

Deep-frozen (liquid nitrogen) tumor tissue samples of ovarian cancer patients were obtained from the biobank of the Department of Obstetrics and Gynecology and the Institute of Pathology (Klinikums rechts der Isar, TU Munich, Germany). Total DNA and RNA were isolated from the tissues using the automated QIAcube sample preparation machine (Qiagen), together with the All Prep DNA/RNA Universal kit (Qiagen) following the manufacturer’s instructions [25].

Concentration and purity of the isolated total RNA samples were spectrophotometrically assessed at 260/230 and 260/280 nm, respectively, using the Nano Drop 2000c spectrophotometer and the Nano Drop 2000/2000c software (Thermo Fisher Scientifc, Wilmington, DE, USA).

Reverse transcription of the isolated RNA for generation of first-strand cDNA was performed using random hexamer primers and the AMV First Strand cDNA Synthesis Kit (Invitrogen, Darmstadt, Germany), following the manufacturer’s instructions. For storage of cDNA samples at − 20 °C, 80 μl RNase-free water were added to each sample (1:5 dilution), resulting in final cDNA concentrations of approximately 10 ng/μl for KLK4 and 5 ng/μl for the patients’ samples.

### Primer designs and quantitative real-time PCR

mRNA expression of the KLK4 biomarker was determined by quantitative RT-PCR using an Agilent MX3005P system (Agilent, Darmstadt, Germany). For this purpose, assays have been established in-house using the ProbeFinder software and the Universal ProbeLibrary (Roche, Penzberg, Germany). Hypoxanthine-Guanine Phosphoribosyl transferase 1 (HPRT1) was used as reference gene, suitable for the assessment of biomarkers in breast and ovarian cancer tissues.

Gene specific primers were designed using the Universal Probe Library Assay Design Center software (Roche). The following primers (Metabion, Martinsried, Germany) and hydrolysis probes from the Universal Probe Library were selected:

HPRT1 (numbers for the location of the primers are according to the NCBI entry NM_000194) (reaction concentrations: 400 nM each). The primers for HPRT: HPRT1-forward (218–241): 5’-TGACCTTGATTTATTTTGCATACC-3’; HPRT1- reverse (300–319): 3’-CGAGCAAGACGTTCAGTCCT-5’; HPRT1-probe: 5’-FAM- GCTGAGGA (reaction concentration: 200 nM); amplicon size: 102 bp.

For KLK4, the assay was optimized to use a TaqMan gene expression assay from ThermoFisher for characterizing KLK4 mRNA expression (assay ID: Hs05041835_s1). The assay detects KLK4 mRNA variants 1 and 2 (NM_001302961.1, NM_004917.4), both encoding the identical, full length KLK4 protein but with differing non-coding sequences.

Threshold cycles (Ct) were employed to calculate the grade of KLK4 mRNA expression compared to the HPRT1 housekeeping gene by relative quantification using the 2exp-ΔΔCt method [30]. Data were statistically analyzed by the SPSS data analysis software (version 20.0; SPSS Inc., Chicago, IL, USA). Standard dilution series were performed to prove appropriate reaction efficiency and sensitivity and to account for the variations and heterogeneity of the different extractions, the reverse transcription reactions and the master mix [31].

### Statistical analysis

Data analyses were carried out by employing the SPSS statistical analysis software (version 20.0; SPSS Inc). The Chi-square test was used for evaluation of the correlations between KLK4 mRNA expression levels and the patients’ clinical characteristics. The Cox univariate and multivariable proportional hazards regression models were built to assess the association of KLK4 mRNA expression levels and clinical parameters with PFS and OS. The multivariable Cox regression analysis included KLK4 mRNA levels and the established clinical parameters like age, ascites fluid volume, and residual tumor mass.

For survival analyses, OS and PFS were used as follow-up end points. Kaplan-Meier survival analysis was used to plot the survival curves and the log-rank test was used to assess the observed differences between Kaplan-Meier PFS and OS curves. In all statistical tests of this study, p values < 0.05 were considered statistically significant.

## Results

### Quantification of KLK4 mRNA expression and its relation to clinical characteristics in tumor tissues of advanced high-grade serous ovarian cancer

**Fig 1:**
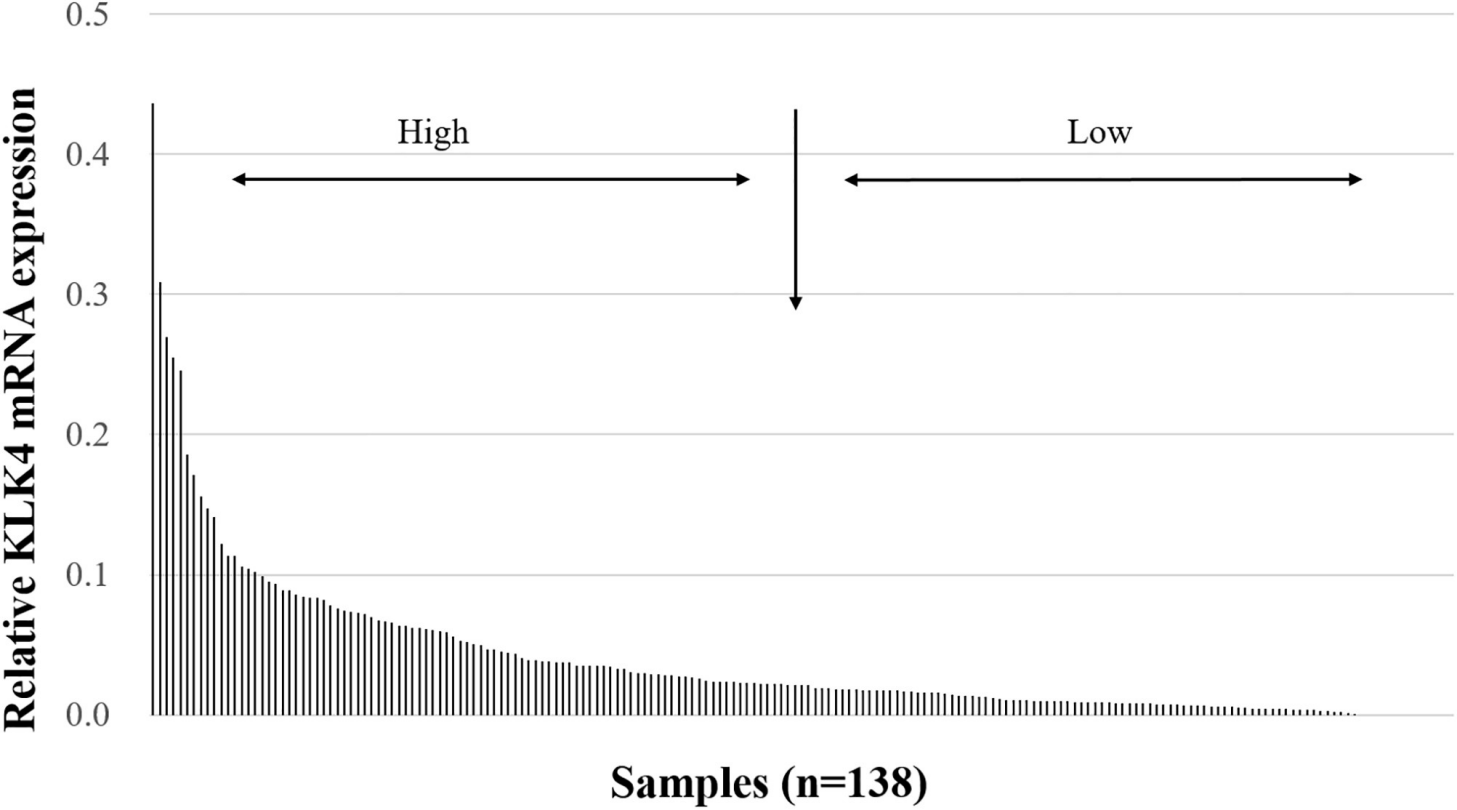
KLK4 mRNA expression levels in tumor tissues of patients afflicted with advanced high-grade serous ovarian cancer (FIGO III/IV). The cumulative histogram depicts relative KLK4 mRNA expression levels (normalized to HPRT mRNA levels). Most samples display very low expression. For further analysis, we categorized the KLK4 mRNA expression levels into low expression versus high expression groups by the 50^th^ percentile (median).

KLK4 mRNA expression levels were assessed in 138 tumor tissues of patients with advanced high-grade serous ovarian cancer (FIGO stage III/IV). The relative KLK4 mRNA levels ranged from 0 to 0.44 (median = 0.019). On the basis of the expression pattern of KLK4 (Fig 1), we categorized the KLK4 mRNA expression in a low-expressing group versus a high-expressing group by the median (50^th^ percentile).

Table 1 shows the correlations of KLK4 mRNA levels with the established clinical parameters, including age, post-operative residual tumor mass, and pre-operative ascites fluid volume. Significant associations were observed between KLK4 mRNA expression and patients’ age as well as pre-operative ascites fluid volume (p = 0.006 and p = 0.042, respectively).

**Table 1:** Association between KLK4 mRNA expression levels and clinical characteristics in patients with advanced high-grade serous ovarian cancer (FIGO stage III/IV).

Clinical parameters	No. of patients	KLK4
		Low/high ^a^
**Age**		**p = 0.006**
**≤ 60 years**	58	37/21
**> 60 years**	80	32/48
**Residual tumor mass**		p = 0.303
**0 mm**	70	38/32
**> 0 mm**	66	30/36
**Ascites fluid volume**		**p = 0.042**
**≤ 500 ml**	78	45/33
**> 500 ml**	53	21/32

Due to missing values, numbers do not always add up to n = 138.

^a^ Categorized into low- and high-expressing groups by median.

### Associations of KLK4 mRNA expression levels as well as clinical parameters with patients’ survival

**Fig 2:**
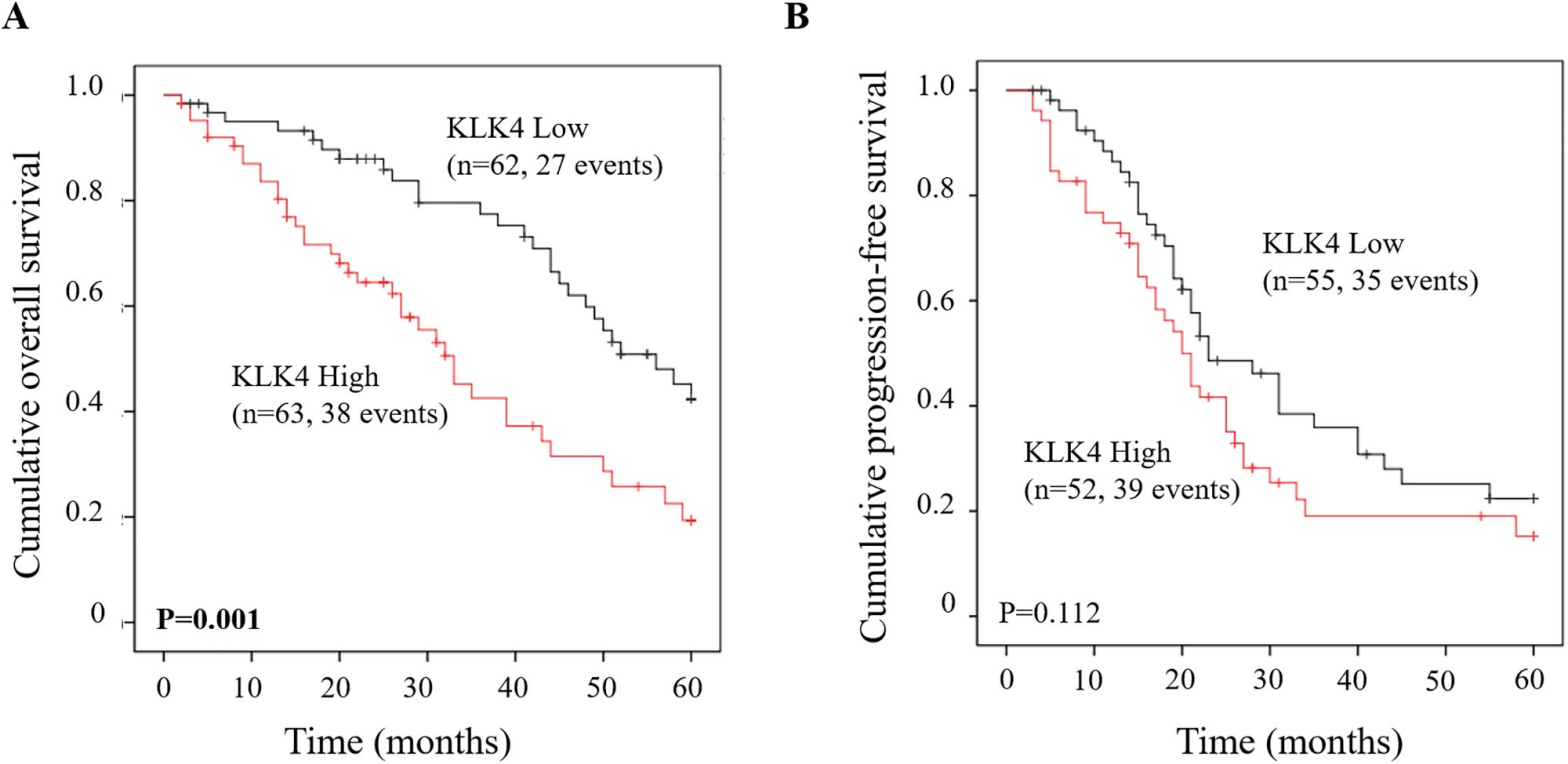
Probability of PFS and OS of patients with advanced high-grade serous ovarian cancer (FIGO III/IV) as stratified by KLK4 mRNA expression levels in primary tumor tissues. Patients with high KLK4 mRNA expression levels display a significantly worse OS rate (Kaplan-Meier analysis, p = 0.001) (A) but not PFS (B), compared to patients with low KLK4 mRNA expression levels.

Table 2 demonstrates the associations of relevant clinical parameters and KLK4 mRNA expression with patients’ 5-year overall survival (OS) and progression-free survival (PFS) by univariate Cox regression analysis. OS data were available for 126 patients, PFS data for 108 patients. Residual tumor mass and ascites fluid volume were confirmed to be univariate predictors for both OS and PFS. Elevated KLK4 mRNA expression was notably linked with shortened OS (hazard ratio [HR] = 2.28, 95% CI = 1.38–3.76, p = 0.001). However, with respect to PFS, no significant association with KLK4 mRNA levels was observed (Table 2). These findings were verified by the respective Kaplan-Meier survival curves again indicating a significant difference between high and low KLK4 expression concerning OS (p = 0.001) but not PFS (Fig 2).

**Table 2:** Univariate Cox regression analysis of KLK4 mRNA expression levels and patients’ survival in advanced high-grade ovarian cancer (FIGO III/IV).

Clinical parameters	OS			PFS		
	No ^a^	HR (95% CI) ^b^	p	No ^a^	HR (95% CI) ^b^	p
**Age**			0.348			0.627
**≤ 60 years**	50	1		43	1	
**> 60 years**	76	1.27 (0.77–2.08)		65	1.12 (0.70–1.79)	
**Residual tumor mass**			**< 0.001**			**< 0.001**
**0 mm**	64	1		59	1	
**> 0 mm**	60	3.76(2.18–6.48)		49	2.53 (1.60–4.02)	
**Ascites fluid volume**			**0.011**			**0.018**
**≤ 500 ml**	72	1		63	1	
**> 500 ml**	47	1.93 (1.16–3.18)		39	1.78 (1.10–2.87)	
**KLK4 mRNA** ^c^			**0.001**			0.121
**low**	62	1		55	1	
**high**	63	2.28(1.38–3.76)		52	1.44(0.91–2.78)	

Significant p-values (p < 0.05) are indicated in bold.

^a^ Number of patients; due to missing values, numbers do not always add up to n = 126 (OS) and n = 108 (PFS).

^b^ HR: hazard ratio (CI: confidence interval) of univariate Cox regression analysis.

^c^ Dichotomized into low and high levels by median.

**Fig 3:**
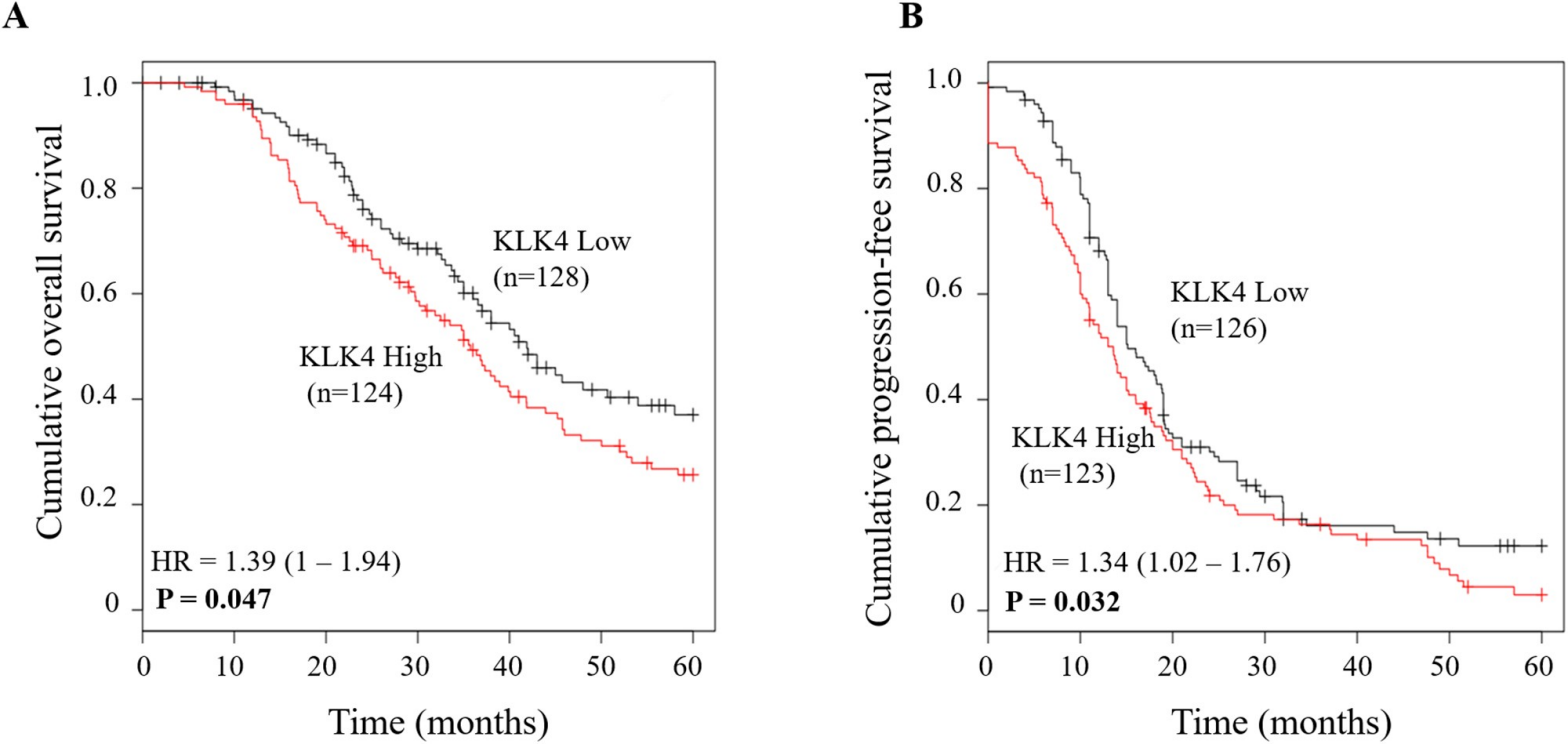
Validation of significant associations between KLK4 mRNA expression and patient outcome in a publicly available Affymetrix microarray data set. The microarray data set analyzed for KLK4 mRNA expression (probe ID 1555737_a_at) was from The Cancer Genome Atlas (TCGA). The patients were selected for serous histological type, advanced stage (FIGO III/IV), high-grade (grade 3), platinum-containing chemotherapy, and 5 years’ follow-up. This selection resulted in a patient cohort encompassing 252 patients for analysis of the association with OS (A) and 249 patients for PFS (B), respectively.

To validate the results obtained for KLK4, we used the Kaplan-Meier Plotter to perform an *in silico* analysis of publicly available Affymetrix-based mRNA data from ovarian cancer patients [32]. For this, we applied the data set from The Cancer Genome Atlas (TCGA) and selected for high-grade (grade 3) serous ovarian cancer patients in advanced stage (FIGO III+IV), who had received platinum-based chemotherapy. This selection resulted in a patient cohort amounting to 252 patients for analysis of the association with OS and 249 patients for PFS, respectively. Kaplan-Meier analysis (with 5 years follow up) confirmed that, in line with our results, elevated KLK4 mRNA levels were significantly associated with a shortened OS (p = 0.047). In this ovarian cancer cohort, however, also a significant association of elevated KLK4 mRNA levels with PFS was observed (p = 0.032) (Fig 3).

To further evaluate the impact of KLK4 mRNA expression on prognosis, a multivariable analysis was performed, including the clinical parameters age, residual tumor mass, and ascites fluid volume (base model) as well as KLK4 mRNA expression (Table 3). In the base model, residual tumor mass was the only independent indicator both for OS (HR = 3.58, 95% CI = 1.90–6.74, p < 0.001) and PFS (HR = 2.36, 95% CI = 1.38–4.05, p = 0.002). Upon addition to the base model, KLK4 mRNA expression displayed statistical significance for OS (HR = 2.31, 95% CI = 1.27–4.20, p = 0.006).

**Table 3:** Multivariable Cox regression analysis of KLK4 mRNA expression levels and patients survival in advanced high-grade ovarian cancer (FIGO III/IV).

Clinical parameters	OS			PFS		
	No^a^	HR (95% CI) ^b^	p	No ^a^	HR (95% CI) ^b^	p
**Age**			0.470			0.733
**≤ 60 years**	47	1		41	1	
**> 60 years**	69	1.22 (0.72–2.07)		60	0.92 (0.56–1.51)	
**Residual tumor mass**			**< 0.001**			**0.002**
0 mm	63	1		58	1	
> 0 mm	53	3.58 (1.90–6.74)		43	2.36 (1.38–4.05)	
**Ascitic fluid volume**			0.911			0.474
**≤ 500 ml**	71	1		63	1	
**> 500 ml**	45	1.03 (0.58–1.86)		38	1.22 (0.71–2.10)	
**KLK4 mRNA** ^c^			**0.006**			0.284
**low**	58	1		52	1	
**high**	58	2.31 (1.27–4.20)		49	1.32(0.79–2.20)	

The biological marker KLK4 mRNA was added to the base model of clinical parameters: age, residual tumor mass, and ascites fluid volume. Significant p-values (p < 0.05) are indicated in bold.

^a^ Number of patients;

^b^ HR: hazard ratio (CI: confidence interval) of multivariable Cox regression analysis;

^c^ Dichotomized into low and high levels by median.

## Discussion

In the present retrospective study, we analyzed the mRNA expression levels of KLK4 in a homogenous cohort of 138 patients suffering from advanced high-grade serous ovarian cancer (FIGO stage III/IV) by qPCR. Moreover, the impact of KLK4 mRNA expression on clinical outcome was evaluated by univariate and multivariable Cox regression analysis. In previous studies, KLK4 protein was demonstrated to be upregulated in serous ovarian cancer, as compared with the expression in normal ovary tissues [5,27]. Obiezu and co-workers found that KLK4 mRNA was more frequently expressed in advanced-stage and high-grade ovarian cancers, in comparison with patients with early stage and lower grade ovarian cancers, suggesting that elevated KLK4 expression is correlated with more aggressive tumor subtypes [29]. Still, according to the data from The Cancer Genome Atlas (TCGA) (https://www.proteinatlas.org/), the majority of ovarian cancer tissues display low expression levels of KLK4 mRNA. In accordance with these data, in the current study KLK4 mRNA was detectable at low levels in most of the advanced high-grade serous ovarian cancer samples as well (Fig 1).

Our analyses of the correlations of KLK4 mRNA expression with clinical parameters demonstrated that KLK4 mRNA levels are significantly associated with the amount of pre-operative ascites fluid volume in advanced high-grade serous ovarian cancer. A higher proportion of elevated KLK4 expression in tumor tissue was observed in patients with an ascites fluid volume > 500 ml (60%, 32/53; Table 1), compared to those with an ascites fluid volume ≤ 500 ml (42%, 33/78; Table 1), which is in line with previous studies demonstrating high levels of KLK4 protein in the effusion fluid of patients suffering from serous epithelial ovarian cancer [13,33]. Furthermore, these findings are in agreement with former studies showing that elevated KLK4 protein and mRNA levels are both correlated with more invasive and aggressive ovarian cancer phenotypes [13,29]. Therefore, KLK4 may play a similar role like other KLKs in promoting tumor invasiveness and metastasis. Of note, however, Shih and co-workers [19] observed only very low concentrations of KLK4 protein in effusion fluid of ovarian cancer patients. This discrepancy is likely due to the fact that in the study of Shih et al. [19] the secreted KLK levels were measured by ELISA while the current study analyzed gene expression levels by qPCR, suggesting that although KLK4 mRNA is expressed in ovarian cancer cells, secreted KLK4 protein may not be measurable in the extracellular environment because of the detection limit of the ELISA [19].

Both KLK4 mRNA and protein levels have been reported to be a prognostic marker in various types of cancer: in prostate, oral and breast cancer, overexpression of KLK4 protein and mRNA was associated with an unfavorable prognosis [29,34–36]. In contrast, in laryngeal squamous cell carcinoma, KLK4 mRNA was proposed to be a favorable biomarker for prognosis [37]. In case of ovarian cancer, a significant association was observed between elevated KLK4 mRNA and protein expression and poor clinical outcome, including progression-free and overall survival [13,29]. In addition, Xi and co-workers found that KLK4 is related with paclitaxel resistance in ovarian cancer [38]. These findings suggest that KLK4 may mediate adverse effects in ovarian cancer. In line with previous studies [13,29], our current study indicates that high KLK4 mRNA expression is significantly linked with shortened OS in univariate Cox regression analysis, whereas no significant association with PFS was observed (Table 2). Kaplan-Meier analysis further confirmed the unfavorable prognostic power of KLK4 mRNA with respect to OS (Fig 2A). In *in silico* analysis using an Affymetrix-based ovarian cancer data set from The Cancer Genome Atlas (TCGA) selected for parameters with the same characteristics as in the analyzed cohort, elevated KLK4 mRNA expression was shown to be significantly associated with both shorter OS (p = 0.047) as well as PFS (p = 0.032). Considering the trend lines in Kaplan-Meier analysis for the association of KLK4 levels with PFS in our analyzed patient cohort (Fig 2B) it is tempting to speculate that the lack of a significant association with PFS is due to the rather low patient numbers. Thus, if more patients were enrolled, KLK4 may also display predictive power with respect to PFS.

In multivariable analysis, our data showed that apart from residual tumor mass, KLK4 mRNA was an independent prognostic indicator for poor OS (p = 0.006) in advanced high-grade serous ovarian cancer. Consistent with our findings, Obiezu and co-workers found that KLK4 mRNA is an independent unfavorable prognostic biomarker on OS in the subgroup of high grade ovarian cancer patients (n = 88) as well [29].

The results of recent studies indicate that KLKs are involved in a multitude of physiological processes and cancer progression [39,40]. Whereas in the normal ovary only KLK10 and to a lesser extent KLK11 and KLK14 are expressed, most of the members of the KLK family are considerably upregulated in ovarian cancer (reviewed in [41]). Strong expression is seen in the case of KLK5-8, 10 and 11, followed by moderate expression of KLK1, 13, and 14. The other KLKs, including KLK4, are found to be weakly expressed [41]. Thus, it seems very unlikely that KLK4 is involved in major extracellular proteolysis such as degradation of proteins of the extracellular matrix (ECM). However, KLK4 could still fulfill important regulatory roles via activation of signaling molecules or modulation of tumor-associated pathways. KLK4 may, for example, affect ECM remodeling by modulating the tumor-associated urokinase-type plasminogen activator (uPA) / urokinase-type plasminogen activator receptor (uPAR) system activity, i.e. by activation of the pro-form of uPA or cleaving its receptor uPAR [42]. Moreover, KLK4 was reported to exert its tumor biological effects through activating multiple secreted growth factors like insulin-like growth factor (IGF) [43], hepatocyte growth factor/scatter factor (HGFSF) [44,45] and transforming growth factor (TGF-β) [46]. Additionally, several members of the KLK family, including KLK4, can serve as signal molecules controlling cell functions through the protease-activated receptors (PARs), being of concern in the progression of several cancers including ovarian cancer [47]. In line with the proposed signaling functions, we recently demonstrated that KLK4, in combination with KLK5, 6, and 7, regulates gene expression of other tumor-relevant genes such as MSN (encoding moesin), KRT7 and KRT19 (encoding keratins 7 and 19) [48].

Under (patho-)physiological conditions, KLKs often form proteolytic cascades/networks resulting in coordinate regulation of important cellular processes. Therefore, it is not too surprising that we observed indication for coordinate expression of some KLKs such as KLK6/KLK8 (Spearman correlation of mRNA levels: r_s_ = 0.636; [25]), KLK10/KLK11 (r_s_ = 0.647; [26]), or KLK5/KLK7 (r_s_ = 0.568; unpublished results) in advanced high-grade serous ovarian cancer. However, the correlation between mRNA expression of KLK4 and that of other KLK family members, KLK5-11 and KLK13-15, was generally found to be low (with r_s_ values <0.25; data not shown).

Using the TCGA RNA-seq data set for ovarian cancer (https://www.proteinatlas.org/), we also analyzed whether there are any indications for coordinate expression of KLK4 and members of the plasminogen activation system, including the KLK4 substrates uPA and uPAR [42] as well as PAI-1. Whereas uPA, uPAR and PAI-1 are clearly coordinately expressed (displaying r_s_ values between 0.567 and 0.685), only low correlations were observed between KLK4 mRNA levels and those of uPA, uPAR and PAI-1 (r_s_ values <0.25). Still, upon dichotomization of KLK4 mRNA expression levels into low and high (by the median) and analysis for association with uPA, uPAR, and PAI-1 mRNA values, respectively, we observed that—with a considerable overlap—higher KLK4 mRNA levels are significantly associated with higher uPA, uPAR, or PAI-1 mRNA levels (Mann-Whitney test; p<0.001 in all cases). Therefore, it may be of interest to analyze the clinical relevance of combinations of KLK4 and its substrates, e.g. KLK4 high / uPA high versus KLK4 and/or uPA low. By this, it can be examined whether increased expression of both, KLK4 and its substrates, is associated with a worse prognosis of patients. Because KLK4 has been shown to have the potential to activate several members of the KLK family, including KLK1-3, 5, 6, 9, and 11–15 [49], combinatorial analyses may two (or more) KLKs enable a better separation into groups with favorable versus unfavorable prognosis as well.

## Conclusions

In the present study, we found a significant association of elevated KLK4 mRNA expression levels with shortened OS analyzing a homogenous cohort of advanced high-grade serous ovarian cancer patients (FIGO stage III/IV). Strikingly, upon addition of KLK4 mRNA into the multivariable model we showed that KLK4 remains an independent unfavorable predictive biomarker for OS in ovarian cancer. In the context with previous findings indicating an important tumor biological role of KLK4 in regulation of cell proliferation, adhesion, migration, and invasion, KLK4 may represent an attractive novel target for tumor therapy in ovarian cancer.
